# Reoperative Selective Sentinel Lymphadenectomy Combined With Lymphoscintigraphy Is Technically Feasible for Cutaneous Tumors of the Upper Extremity After Radical Dissection of Regional Lymph Node Basins for Breast Cancer

**Published:** 2014-09-12

**Authors:** Edward P. Miranda, Oliver C. Bellevue, Stanley P.L. Leong

**Affiliations:** ^a^Center for Complex Reconstruction, San Francisco, Calif; ^b^School of Medicine, University of California San Francisco; ^c^Center for Melanoma Research and Treatment, Department of Surgery, California Pacific Medical Center, San Francisco, Calif

**Keywords:** lymphadenectomy, lymphoscintigraphy, melanoma, sentinel node, squamous cell carcinoma

## Abstract

**Objective:** The rising incidence of melanoma and the high prevalence of breast cancer have generated a new scientific problem—how do the regional lymph node basins function after radical lymphadenectomy and are lymphatic drainage patterns altered after radical lymphadenectomy? Furthermore, after radical lymphadenectomy, selective sentinel lymphadenectomy is still a technically feasible and valid staging tool in the upper extremity? Thus, our study asks if selective sentinel lymph node dissection is technically feasible after radical lymph node dissection of the regional draining basin of the upper extremity (axilla). **Methods:** Retrospective review of a prospectively maintained database of patients was reviewed to identify patients who had lymphoscintigraphy and sentinel lymph node biopsy of the upper extremity after a radical axillary node dissection procedure. Imaging and pathology results were analyzed. **Results:** Seven patients fulfilling the inclusion criteria were identified. The patients all had either melanoma or invasive squamous cell carcinoma, and sentinel lymph nodes were identified in 6 out of 7 patients. One patient had metastases to 2 sentinel lymph nodes. Alternative drainage pathways were identified in 29% of patients, and 14% of patients had no identifiable drainage basin on lymphoscintigraphy. **Conclusions:** Sentinel lymph node dissection is technically feasible after previous axillary dissection. Lymphoscintigraphy is an important perioperative tool as lymphatic drainage may be altered or not observed as evidenced in 43% of the studied patients. However, when lymphatic drainage is detected by lymphoscintigraphy, pathologically significant sentinel lymph nodes are surgically identifiable.

Selective sentinel lymphadenectomy (SSL) as described by Morton, has become the standard of care for the staging of melanoma and adenocarcinoma of the breast.^1^ It is also being used with increasing frequency in other malignancies such as squamous cell carcinoma and colon cancer. The effectiveness of the technique is so profound that it is the primary regional staging technique for breast cancer and the use of SSL in asymptomatic melanoma patients obviates the need for additional imaging studies at the time of diagnosis.

The rising incidence of melanoma and the high prevalence of breast cancer have generated a new scientific problem—how do the regional lymph node basins function after radical lymphadenectomy? Or restated in more clinical terms, are lymphatic drainage patterns altered after radical lymphadenectomy and is SSL still a technically feasible and valid staging tool? This challenging problem is just beginning to be addressed in canine animal models, which suggest that alternative lymphatic pathways are present in some cases.^2^^,^^3^

Over the last decade, several reports regarding the use of SSL or radical lymphadenectomy for previous axillary surgery have surfaced. Dinan et al^4^ reported the locations of the sentinel lymph nodes (SLNs) of 11 patients who previously had either SSL or radical axillary lymph node dissection (ALND). Unusual drainage patterns were present in 4 patients (36%), including clavicular, posterior cervical, intramammary, and internal mammary locations (all ipsilateral). Sood et al presented 4 selected cases in which SSL for breast cancer was performed in which there was a prior ipsilateral ALND. Alternate sites included ipsilateral intramammary, internal mammary, and contralateral axillary SLNs.^5^ Subsequent to these initial reports, other studies have examined the imaging modality for recurrence of breast cancer and have reported good success in identifying sentinel nodes in patients with previous ALND.^6^^,^^7^

In spite of these reports, the feasibility of SSL for cutaneous tumors of the upper extremity in which a previously radically dissected axilla with a predicted regional drainage pattern into the axilla has never been explored. The purpose of this study was to examine lymphatic drainage patterns of the upper extremity in patients with previous ALND and determine if lymphoscintigraphy is an effective imaging modality for this indication.

## MATERIALS AND METHODS

### Patients

Patients who previously had an axillary lymph node dissection and had a cutaneous tumor indicating SSL with an anticipated lymphatic drainage pattern to the dissected axilla were identified. Demographic data and medical histories were collected from each of the patients.

### Lymphoscintigraphy

Each of these patients underwent preoperative lymphoscintigraphy technetium sulfur colloid either the day before, or the morning of SSL. Approximately 750uCi of ^99^Tc sulfur colloid was administered intradermally at the site of the primary tumor. Subsequent scintigraphy was performed and dynamic and static images recorded.

### Selective sentinel lymphadenectomy

Each patient underwent wide local excision of the primary tumor sites. Subsequent SSL was directed by images provided by the lymphoscintigram. SLNs were identified using a handheld gamma counter (NeoProbe, Devicor Medical Products, Inc, Cincinatti, OH). All lymph nodes with *in vitro* gamma counts that were above 10% of the most radioactive node identified were excised and categorized as SLNs.

### Pathology

The wide local excisions and the SLNs were submitted to pathology. SLNs underwent serial sectioning and hematoxylin and eosin staining. For patients with melanoma, S-100 proteins and human melanoma black-45 immunohistochemical staining was also performed. Positive SLNs were ones that had tumor cells on haematoxalin and eosin staining or demonstrated S-100 proteins or human melanoma black-45 staining.

## RESULTS

### Patients

Seven patients were identified who had melanoma with a thickness of more than 0.8 mm or severe squamous cell carcinoma (indicating SSL) and previously had an ALND. All patients had a predicted lymphatic drainage pattern that would include the axilla. Four had modified radical mastectomies ipsilateral to the cutaneous tumor site, 2 had bilateral radical mastectomies, and the 1 had a lumpectomy and ALND. Patient and tumor characteristics are presented in Table 1.

### Lymphoscintigraphy

Six of the 7 patients successfully underwent lymphoscintigraphy. In one case, SLNs were not identified. This success rate is similar to what has been published in other related studies.^6^ The ipsilateral axilla was the major regional lymphatic draining basin in 6 of the cases. Details of the lymphoscintigram are given in Table 2.

### Selective sentinel lymphadenectomy

In 6 of 7 patients who had successful lymphoscintigraphy, at least 1 SLN was identified in each of the patients. The identified SLNs correlated to the locations predicted by the lymphoscintigraphy. One of the patients had metastases to 2 SLNs, the others had no evidence of regional disease (Table 2).

## DISCUSSION

The primary purpose of this study was to demonstrate that SSL is technically feasible for patients whose predicted drainage basin had previously undergone radical lymphadenectomy. All 7 patients successfully underwent the procedure with the detection of radioactive lymph nodes. The presence of metastatic disease in 2 SLNs from 1 of the 7 patients is proof of principle—that in the presence of potentially altered lymphatics the SLN concept is valid in at least that radiotracer and metastases can follow the same pattern. It also demonstrates that careful, radioguided dissection is safe and can identify residual lymph nodes in a previously dissected axilla. What this study does not address is whether or not SSL has a similar reliability for staging. That 5 of the 6 patients with identified SLNs had SLNs negative for metastasis is certainly not unexpected; however, this study is underpowered to generate any reliable conclusion as to whether or not the false-negative rate should be similar to that which is reported in the literature.

The preoperative hypothesis was that lymphoscintigraphy would either reveal alternative lymphatic drainage pathways and basins (eg, supraclavicular nodes) or drainage via residual axillary nodes. The findings of both alternative pathways (eg, internal mammary nodes) as well as drainage to previously dissected axillae brings up several important considerations—including whether or not there is a “standard” postaxillary dissection lymph node pattern and if under these conditions SSL is accurate in predicting regional nodal metastasis.

The lymphatic drainage to the axilla after dissection has not been fully studied. Most investigation has centered on patients with breast cancer–related lymphedema. Stanton et al^8^^,^^9^ have studied (via lymphoscintigraphy) the drainage of the upper extremity after ALND in the subset of patients with postoperative lymphedema. In one study, 24 patients with postoperative lymphedema without hand edema were injected with radiolabeled IgG in the hand; the nonoperated arm was used as the control.^10^ No axillary lymph nodes were detected on the edematous, operated side. Furthermore, it appeared that there was increased lymphatic drainage along alternative routes, including the superficial tissues of the forearm and upper arm when compared with controls.

In a different study, fluorescence microlymphography revealed superficial lymphangiogenesis in patients with lymph edema.^11^ When contrasted with controls who had undergone ALND for breast cancer *not* complicated lymphedema, new lymphatics were shown to form only in those *with* lymphedema. This is confirmed by another study that demonstrated that dermal lymphangiogenesis occurs only in patients with postdissection lymphedema. Unfortunately, these and other studies do not provide a comprehensive understanding of axillary drainage after ALND, particularly in those patients not complicated by lymphedema. However, it underscores the potential for alternative drainage patterns to the axilla in the presence of lymphedema.

Of all the observations mentioned earlier, probably the most important is that lymphoscintigraphy is an important tool for preoperative planning. With a multitude of possible drainage patterns, the data suggest that the use of isosulfan blue alone may be inadequate. Even radiocolloid injection and use of only the handheld gamma counter may similarly be inadequate as precise visualization of the SLN is not possible—potentially leading to incision over a “hot spot” in the supraclavicular area only to find that the node detected was high (level III) in the axilla. The opposite (inadvertently opening the previously dissected axilla) is possible and is potentially worse—risking injury to the vital structures in the axilla scarred by previous ALND. Lymphatic mapping avoids these problems by giving a pictorial representation of the SLN's anatomic position in several planes.

In the patients presented here, alternative drainage was present in 2 of the 7 patients. Lymphatic mapping was extremely useful for preoperative planning of all cases. It would be interesting to know whether or not lymphedema was present in any of these patients, but because of the retrospective nature of this study, these data were not available.

Although the patient numbers in this study are limited, SSL is demonstrated to be feasible for patients whose cancer has previously been treated with ALND. In these cases where the dissected axilla is an anticipated drainage basin, alternative lymphatic drainage pathways should be considered. Preoperative lymphoscintigraphy can be helpful in delineating the location(s) of the SLNs in these complicated cases and can guide the surgical plan. It is not clear from our study if the lymphatic drainage through a previously dissected axilla represents de novo lymphatic formation or residual lymph channels. There is evidence of lymphatic growth in humans following ALND (Stanton et al) with lymphedema. Although the complex interplay of cytokines responsible for lymphangiogenesis has been well defined in animal models, that understanding has yet been translated into human medicine.^12^ In patients with previous ANLD, we cannot exclude either the possibility of lymphangiogenesis or of residual lymphatics. Unraveling the complexity of these drainage patterns in patients with previous ALND requires lymphoscintigraphy as a helpful means of identifying SLNs. Additional study will be needed to further validate these conclusions.

## Figures and Tables

**Table 1:** Patient and tumor characteristics

Patient number	Years between previous ALND and SSL	Cutaneous tumor location	Cutaneous tumor type	Primary procedure for breast cancer
1	3	Right posterior shoulder	Malignant melanoma, 1.05 mm thick, Clark level IV	Right partial mastectomy and ALND
2	4	Left forearm, extensor surface	Malignant melanoma, 1.5 mm thick, Clark level IV	Left modified radical mastectomy and ALND
3	7	Left forearm, extensor surface	Malignant melanoma, 2.2 mm thick, Clark level IV	Left modified radical mastectomy and ALND
4	15	Left lower, anterior chest wall	Malignant melanoma, >5 mm thick, Clark level IV	Left modified radical mastectomy and ALND
5	6	Right 2^nd^ and 3^rd^ digits	Squamous cell carcinoma	Bilateral modified radical mastectomies and ALND
6	6	Right upper back	Malignant melanoma, 1.0 mm thick, Clark level III	Right modified radical mastectomy
7	Unknown (remote)	Right upper arm/deltoid	Malignant melanoma, 1.9 mm thick	Bilateral modified radical mastectomies and ALND

ALND indicates axillary lymph node dissection; SSL, selective sentinel lymphadenectomy.

**Table 2:** Details of lymphoscintigram, surgery, and subsequent pathology*

Patient number	Basin of drainage	Number of nodes detected via lymphoscintigraphy	Number of nodes removed	Pathology
1	Right supraclavicular and right axilla	3	2 supraclavicular, 1 axillary	Negative
2	Left axilla	6	8	Negative
3	None	0	0	0
4	Left axilla	3	3	2 Positive
5	Right axilla and inferomedial clavicular area	4	3 axillary, 1 inferomedial clavicular area	Negative
6	Right axilla	2	2	Negative
7	Right axilla	1	2	Negative

*Of note, lymphoscintigraphy failed to reveal any lymph nodes in patient 3, therefore sentinel lymph node biopsy was not performed.
